# The accuracy and influencing factors of Doppler echocardiography in estimating pulmonary artery systolic pressure: comparison with right heart catheterization: a retrospective cross-sectional study

**DOI:** 10.1186/s12880-022-00806-5

**Published:** 2022-05-16

**Authors:** Guang-jie Lv, Ai-li Li, Xin-cao Tao, Ya-nan Zhai, Yu Zhang, Jie-ping Lei, Qian Gao, Wan-mu Xie, Zhen-guo Zhai

**Affiliations:** 1 Department of Cardiology, China-Japan Friendship Hospital, Beijing, 100029 China; 2 Department of Pulmonary and Critical Care Medicine, Center of Respiratory Medicine, China-Japan Friendship Hospital, Beijing, 100029 China; 3 National Clinical Research Center for Respiratory Diseases, Beijing, 100029 China; 4 Institute of Clinical Medical Sciences, China-Japan Friendship Hospital, Beijing, 100029 China

## Abstract

**Background:**

Noninvasive assessment of pulmonary artery systolic pressure by Doppler echocardiography (sPAP_ECHO_) has been widely adopted to screen for pulmonary hypertension (PH), but there is still a high proportion of overestimation or underestimation of sPAP_ECHO_. We therefore aimed to explore the accuracy and influencing factors of sPAP_ECHO_ with right heart catheterization (RHC) as a reference.

**Methods:**

A total of 218 highly suspected PH patients who underwent RHC and echocardiography within 7 days were included. The correlation and consistency between tricuspid regurgitation (TR)-related methods and RHC results were tested by Pearson and Bland–Altman methods. TR-related methods included peak velocity of TR (TR Vmax), TR pressure gradient (TR-PG), TR mean pressure gradient (TR-mPG), estimated mean pulmonary artery pressure (mPAP_ECHO_), and sPAP_ECHO_. With mPAP ≥ 25 mm Hg measured by RHC as the standard diagnostic criterion of PH, the ROC curve was used to compare the diagnostic efficacy of sPAP_ECHO_ with other TR-derived parameters. The ratio (sPAP_ECHO_–sPAP_RHC_)/sPAP_RHC_ was calculated and divided into three groups as follows: patients with an estimation error between − 10% and + 10% were defined as the accurate group; patients with an estimated difference greater than + 10% were classified as the overestimated group; and patients with an estimation error greater than − 10% were classified as the underestimated group. The influencing factors of sPAP_ECHO_ were analyzed by ordinal regression analysis.

**Results:**

sPAP_ECHO_ had the highest correlation coefficient (r = 0.781, *P* < 0.001), best diagnostic efficiency (AUC = 0.98), and lowest bias (mean bias = 0.07 mm Hg; 95% limits of agreement, − 32.08 to + 32.22 mm Hg) compared with other TR-related methods. Ordinal regression analysis showed that TR signal quality, sPAP_RHC_ level, and pulmonary artery wedge pressure (PAWP) affected the accuracy of sPAP_ECHO_ (*P* < 0.05). Relative to the good signal quality, the OR values of medium and poor signal quality were 0.26 (95% CI: 0.14, 0.48) and 0.23 (95% CI: 0.07, 0.73), respectively. Compared with high sPAP_RHC_ level, the OR values of low and medium sPAP_RHC_ levels were 21.56 (95% CI: 9.57, 48.55) and 5.13 (95% CI: 2.55, 10.32), respectively. The OR value of PAWP was 0.94 (95% CI: 0.89, 0.99). TR severity and right ventricular systolic function had no significant effect on the accuracy of sPAP_ECHO_.

**Conclusions:**

In this study, we found that all TR-related methods, including sPAP_ECHO_, had comparable and good efficiency in PH screening. To make the assessment of sPAP_ECHO_ more accurate, attention should be paid to TR signal quality, sPAP_RHC_ level, and PAWP.

## Background

Right heart catheterization (RHC) is recognized as the gold standard for measuring pulmonary artery pressure, but its invasiveness limits its general applicability. Doppler echocardiography (DE) can noninvasively assess pulmonary artery pressure by peak velocity of tricuspid regurgitation (TR Vmax) and its derived parameters, including TR pressure gradient (TR-PG), TR mean pressure gradient (TR-mPG), estimated mean pulmonary artery pressure (mPAP_ECHO_), and pulmonary artery systolic pressure (sPAP_ECHO_). The current guidelines recommend TR Vmax to avoid additional error in the estimated right atrial pressure (RAP) [1]. Furthermore, mPAP has been found to be superior to TR Vmax in identifying pulmonary hypertension (PH) [2]. As the most well-adopted approach in PH screening, sPAP_ECHO_ has also been shown to be a reliable method [3]; however, it has not yet been examined whether sPAP_ECHO_ is superior to other parameters in determining the probability of PH. sPAP_ECHO_ can also provide valuable information for evaluating treatment response and even predicting prognosis [4, 5]; however, there is still a high proportion of overestimation or underestimation of sPAP_ECHO_ [6]. To evaluate PH patients’ condition appropriately and avoid too invasive examination, we need to understand situations in which sPAP_ECHO_ is under/overestimated. Based on clinical experience and review of previous literature, we assumed that right ventricular systolic function, pulmonary artery pressure level, TR severity, and signal quality would affect the accuracy of sPAP_ECHO_. In addition, as an important parameter to distinguish pre- and post-capillary PH, pulmonary artery wedge pressure (PAWP) was also included in the analysis to examine whether there would be any difference in the accuracy of sPAP_ECHO_. Therefore, the first aim of this study was to compare the efficiency of sPAP_ECHO_ and other parameters in PH screening, while the second aim was to find influencing factors that account for the inaccuracy of sPAP_ECHO_.

## Methods

Between October 2015 and October 2020, a total of 430 patients admitted to our center with known or suspected PH were evaluated. The inclusion criteria were age ≥ 18 years and the interval between echocardiography and RHC ≤ 7 days. The exclusion criteria were as follows: lack of TR, pulmonary artery stenosis or right ventricular outflow tract stenosis, poor image quality not suitable for analysis, ventricular septal defect, or patent ductus arteriosus. Patients’ demographic and clinical data were obtained from the electronic medical records. The institutional review board of the China–Japan Friendship Hospital waived the need for written informed consent as the study involved the retrospective analysis of clinically acquired data. The data underlying this article will be shared upon a reasonable request to the corresponding author.

### Clinical data

Baseline assessment of the eligible patients included WHO functional class, the level of N-terminal pro B-type natriuretic peptide (NT-proBNP), and a 6-min walk test (6MWT).

### RHC

Hemodynamic measurements were performed with a 7F Swan-Ganz catheter Philips Allura X-PER FD20 flat-plate angiography system (Baxter Inc.). The system was zeroed and referenced at patients’ heart level as previously described [7]. Right atrial pressure (RAP), pulmonary systolic artery pressure (sPAP_RHC_), and PAWP were recorded at end-expiration at baseline over at least three heart cycles. Cardiac output (CO) was obtained using Fick’s method. Pulmonary vascular resistance (PVR), cardiac index, stroke volume, pulse pressure, and diastolic pressure gradient were calculated using standard formulas. Pulmonary artery pressure was classified into low, medium, and high levels according to the tertiles of sPAP_RHC_.

### Echocardiography

**Fig. 1:**
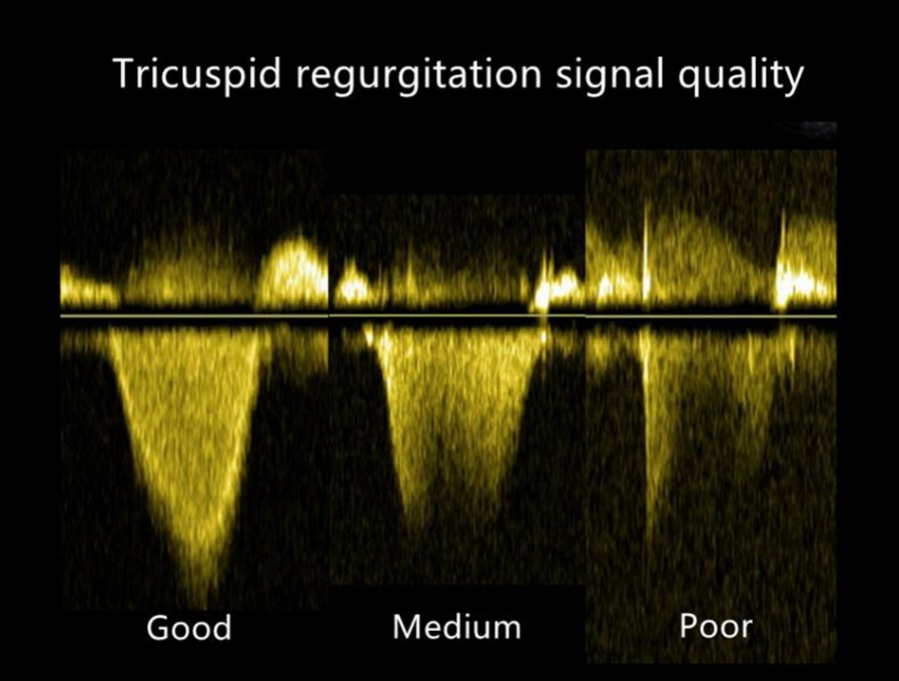
Classification of the TR signal quality using continuous-wave Doppler. Good signal quality, complete envelope; Medium signal quality, partial envelope; Poor signal quality, unreliable envelope or no signal

Echocardiographic images were acquired using a GE Vivid E95 machine (GE Healthcare, General Electric Healthcare) equipped with M5S phased-array transducers. Analysis was performed independently by two blinded investigators using EchoPAC software (GE Healthcare version 201). Two-dimensional echocardiography and Doppler echocardiography (DE) were performed based on current guidelines. TR-PG was calculated from the TR Vmax obtained from continuous-wave Doppler by the simplified Bernoulli equation: TR-PG = 4 (TR Vmax)^2^. TR-mPG was obtained by tracing the time–velocity integral of TR. sPAP_ECHO_ and mPAP_ECHO_ were calculated by adding the estimated RAP to TR-PG and TR-mPG, respectively. RAP was divided into three categories (3, 8, and 15 mm Hg) based on the inferior vena cava (IVC) diameter and its respiratory variation [1]. The ratio (sPAP_ECHO_–sPAP_RHC_)/sPAP_RHC_ was calculated and divided into three groups as follows: patients with an estimation error between − 10% and + 10% were defined as the accurate group; patients with an estimated difference greater than + 10% were classified as the overestimated group; and patients with an estimation error greater than − 10% were classified as the underestimated group. The severity of TR was classified into three grades by comprehensively evaluating the regurgitation jet area and vena contracta (VC) width. The mild group was defined as jet area < 5 cm^2^, VC TR ≤ 3 mm; the moderate group as jet area 5–10 cm^2^, 3 mm < VC TR < 7 mm; and the severe group as jet area > 10 cm^2^, VC TR ≥ 7 mm. TR signal quality was classified into three types according to the extension of the signal for more than half of the systole and well-defined border. Good signal quality was defined as the one that met both criteria. Medium signal quality met only one of these criteria, while poor signal quality did not meet any of the criteria [8] (Fig. 1). RV systolic function was assessed using multiple parameters, including RV wall thickness (RV WT), tricuspid annular plane systolic excursion (TAPSE), systolic annular tissue velocity of the lateral tricuspid annulus (S’), and RV fractional area change (FAC). All of these parameters were repeatedly measured and averaged. To determine the reproducibility of sPAP_ECHO_ measurements, a total of 34 randomly selected examinations were analyzed twice by the first investigator at a 1-week interval and once by the second investigator.

### Statistical analysis

Standard statistical software (SPSS version 26 for Windows, SPSS, Chicago, IL, USA) was used for the statistical analysis. Data are expressed as mean ± standard deviation for quantitative variables with normal distribution, or as median (interquartile range) for variables not complying with normal distribution. The correlation and consistency between TR-derived parameters and RHC results were tested by Pearson and Bland–Altman methods. With mPAP ≥ 25 mm Hg measured by RHC as the standard diagnostic criterion of PH, a receiver operating characteristic (ROC) curve was used to compare the diagnostic efficacy of sPAP_ECHO_ and other TR-related methods. The influencing factors of sPAP_ECHO_ were analyzed by ordinal regression analysis. The intraclass correlation coefficient was used to determine inter- and intra-observer reproducibility for sPAP_ECHO_ from 34 randomly selected patients using an identical cine-loop for each view. For all statistical tests, a *P* value < 0.05 was used to indicate significance.

## Results

### Patients’ characteristics

**Fig. 2:**
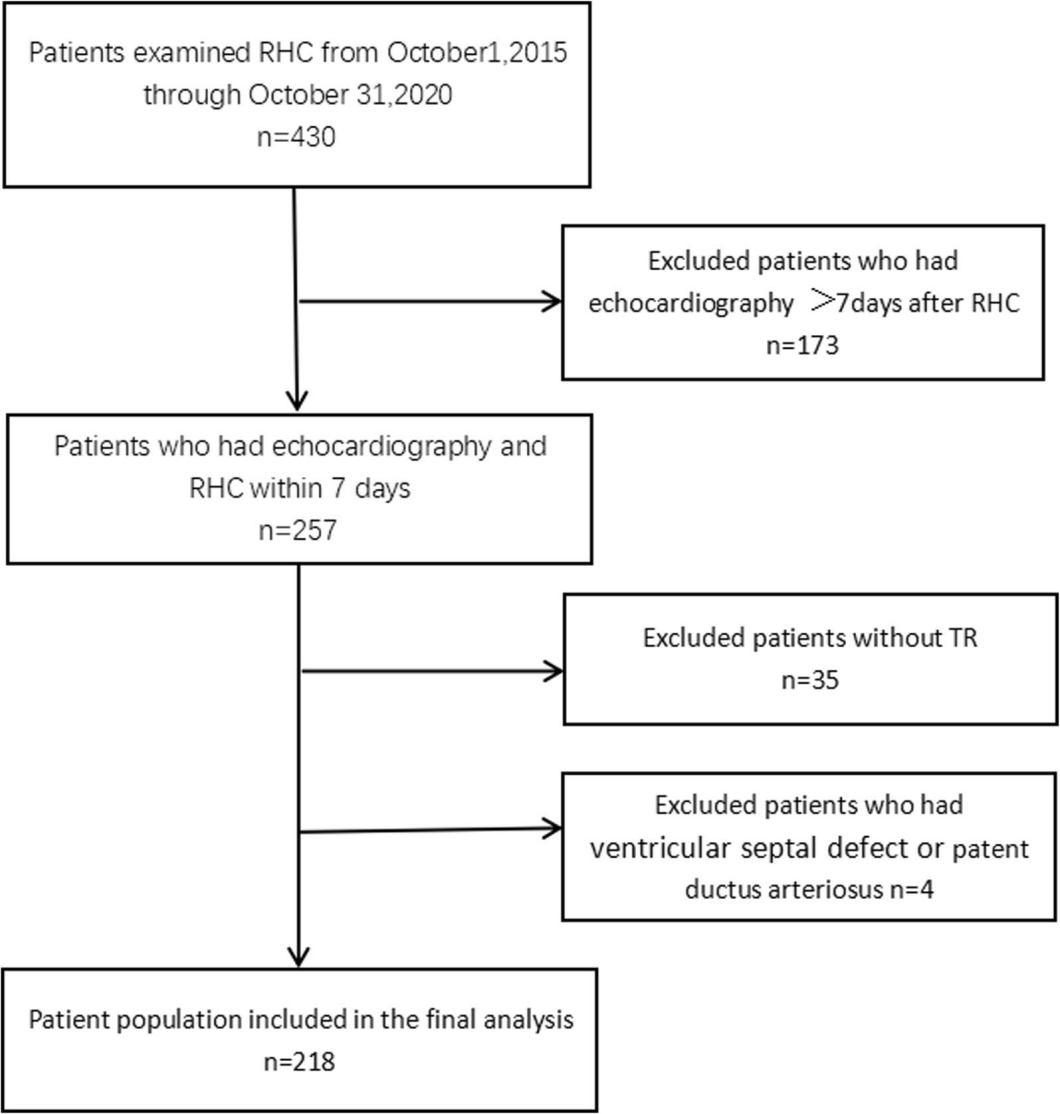
Flow chart of patient screening

A total of 218 patients were finally identified and analyzed, as shown in Fig. 2. Baseline demographic and clinical characteristics are provided in Table 1. The mean age of the patients was 50.9 ± 13.3 years; 40.3% of them were men; 197 (90.4%) patients had PH. None of the patients experienced major cardiac events between DE and RHC examinations. Table 2 lists the DE and RHC variables grouped by estimated accuracy.

**Table 1:** Clinical and demographic characteristics

Variables	Value
Age (years)	50.9 ± 13.3
Males (%)	90 (41.3)
BMI	1.67 (1.57, 1.84)
Systolic BP (mmHg)	120 (108, 132)
Diastolic BP (mmHg)	77 (70, 87)
Heart rate (bpm)	76 (68.65, 80)
Interval between TTE and RHC, days	2.5 (1, 5)
NT-pro BNP (pg/ml)	451 (175, 1043)
6 M WT (m)	365.5 ± 104.6
WHO functional class	
I Class (%)	20 (9.2)
II Class (%)	93 (42.7)
III Class (%)	89 (40.8)
IV Class (%)	16 (7.3)
PH (n)	197 (90.4%)
Idiopathic, heritable, drug and toxic induced	37
Associated with Connective tissue disease	25
Portal hypertension	2
Congenital heart disease	8
PH due to left heart disease	6
PH due to lung disease and/or hypoxia	6
Chronic thromboembolic PH	95
PH with unclear and/or multifactorial mechanisms	13
Pulmonary veno-occlusive disease and/or pulmonary capillary haemangiomatosis	5
Non-PH (n)	21 (9.6%)

Values are presented as mean ± SD, median (IQR), or n (%)

*BMI* body mass index; *TTE*, transthoracic echocardiography; *RHC* right heart catheterization; *BP* blood pressure; *NT-pro BNP* N-terminal pro B-type natriuretic peptide; *6 M WT* 6-min walk test; *PH* pulmonary hypertension

**Table 2:** Univariable and multivariable ordered analysis for accuracy of sPAP_ECHO_

Variables	Overestimation (n = 79)	Accurate (n = 81)	Underestimation (n = 58)	Univariable analysis		Multivariable analysis	
				*P*	OR (95% CI)	*P*	OR (95% CI)
*Echocardiographic parameters*							
RAD (mm)	49.1 ± 10.2	49.9 ± 11.1	49.4 ± 10.0	0.816	0.997 (0.974, 1.021)		
RVDD (mm)	45.4 ± 7.3	46.2 ± 7.6	46.5 ± 6.5	0.323	0.984 (0.952,1.016)		
RV WT (mm)	5.3 ± 1.5	5.4 ± 1.4	5.8 ± 1.6	0.055	0.845 (0.712,1.003)		
TAPSE(mm)	16.8 ± 3.9	16.3 ± 3.4	15.9 ± 3.7	0.110	1.057 (0.988,1.130)		
FAC (%)	30.6 ± 9.3	29.8 ± 8.3	27.7 ± 7.6	0.064	1.029 (0.998,1.061)		
TR severity				0.546			
Mild	44 (20.2%)	51 (23.5%)	36 (16.5%)	0.944	1.031 (0.441,2.408)		
Moderate	28 (12.8%)	22 (10.1%)	16 (7.3%)	0.480	1.386 (0.560,3.431)		
Severe	7 (3.2%)	8 (3.7%)	6 (2.8%)				
TR signal quality				0.020			
Good	53 (24.3%)	49 (22.5%)	25 (11.5%)				
Medium	23 (10.6%)	26 (11.9%)	27 (12.4%)	0.017	0.525 (0.309,0.892)	0.000	0.258 (0.138,0438)
Poor	3 (1.4%)	6 (2.8%)	6 (2.8%)	0.055	0.375 (0.138,1.020)	0.013	0.233 (0.074,0.734)
*Catheterization parameters*							
sPAP_RHC_ level				0.000			
Low	43 (19.7%)	25 (11.5%)	2 (0.9%)	0.000	15.574 (7.563,31.961)	0.000	21.561 (9.574,48.554)
Medium	30 (13.8%)	27 (12.4%)	17 (7.9%)	0.000	5.279 (2.752.10.125)	0.000	5.125 (2.545,10.321)
High	6 (2.8%)	29 (13.3%)	39 (17.9%)				
sPAP_RHC_ (mmHg)	60.5 ± 19.6	74.4 ± 23.1	92.4 ± 23.1	0.000	0.958 (0.947,0.970)		
RAP (mmHg)	2.2 ± 4.2	3.3 ± 4.7	5.2 ± 6.1	0.002	0.921 (0.875,0.970)		
PVR (Wood Units)	8.4 ± 5.9	11.2 ± 6.2	14.0 ± 8.5	0.000	0.912 (0.878,0.947)		
PAWP (mmHg)	6.9 ± 5.2	7.5 ± 5.2	10.1 ± 7.0	0.003	0.932 (0.889,0.977)	0.018	0.939 (0.892,0.989)
mPAP (mmHg)	38.6 ± 35.6	43.3 ± 14.7	53.9 ± 15.5	0.000	0.961 (0.944,0.978)		
*Clinical parameters*							
6 M WT (m)	370.9 ± 105.7	355.9 ± 123.0	367.8 ± 84.1	0.868	1.000 (0.996,1.005)		
WHO functional class				0.907			
I	5 (2.3%)	10 (4.6%)	5 (2.3%)	0.805	0.858 (0.256,2.880)		
II	38 (17.4%)	29 (13.3%)	26 (11.9%)	0.745	1.176 (0.443,3.127)		
III	30 (13.8%)	37 (17.0%)	22 (10.1%)	0.919	1.052 (0.395,2.805)		
VI	6 (2.8%)	5 (2.3%)	5 (2.3%)				

*RAD* right atrial diameter; *RVDD* right ventricle diastolic diameter; *RV WT* right ventricle wall thickness; *TAPSE* tricuspid annular plane systolic excursion; *RV FAC* right ventricle fractional area change; *RAP* right atrial pressure; *PVR* pulmonary vascular resistance; *PAWP* pulmonary artery wedge pressure; *mPAP* mean pulmonary artery pressure; *6 M WT* 6-min walk test

### Observer variability of sPAP_ECHO_ estimation

The intraclass correlation coefficient for interobserver reproducibility of sPAP_ECHO_ was 0.988 (95% CI: 0.977–0.994), and the intraclass correlation coefficient for intraobserver reproducibility of sPAP_ECHO_ was 0.992 (95% CI, 0.984–0.996).

### Association between invasively determined parameters and TR-derived parameters

**Fig. 3:**
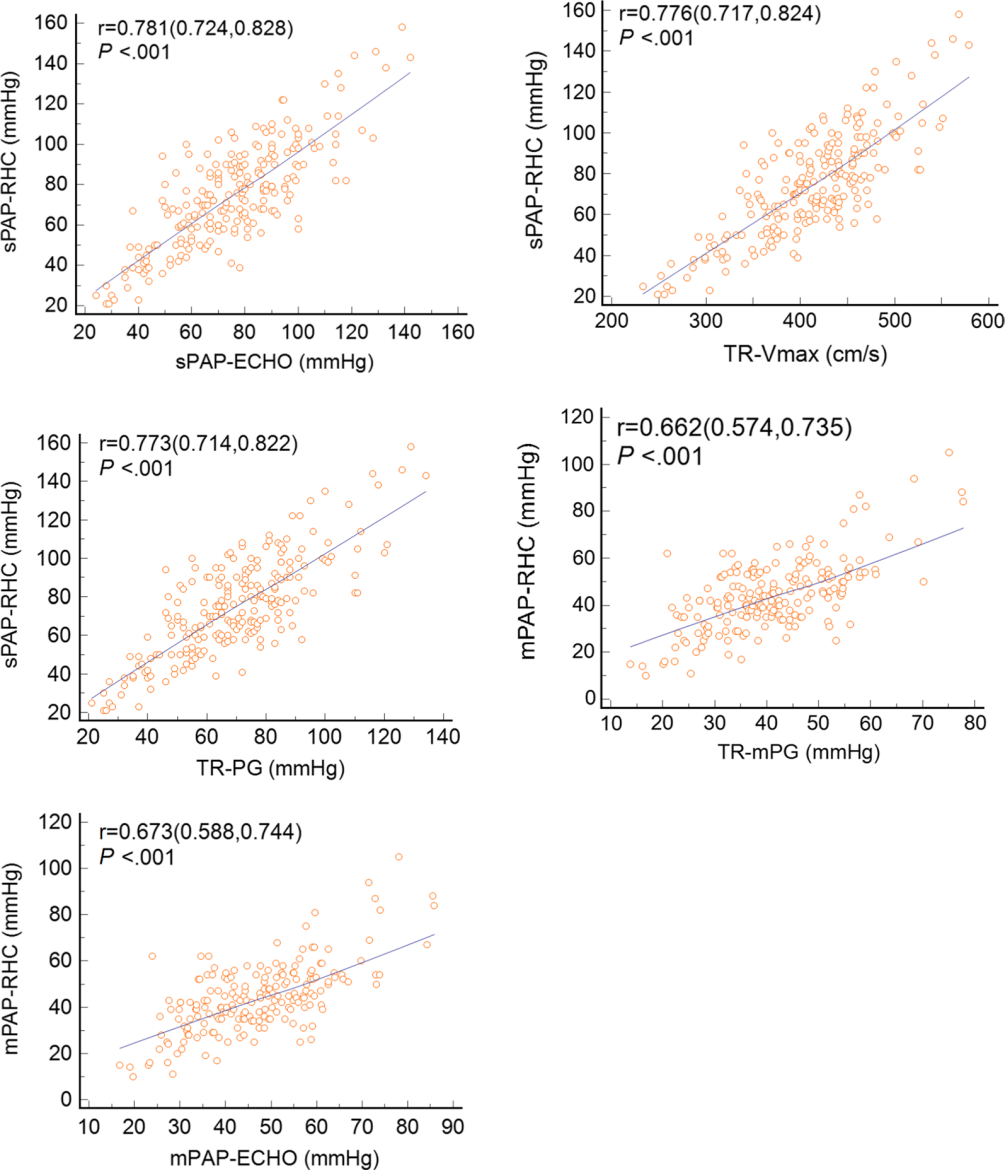
Correlation of invasively determined parameters with TR derived parameters. pearson’s rank correlation coefficients are presented with 95% CI in brackets

**Fig. 4:**
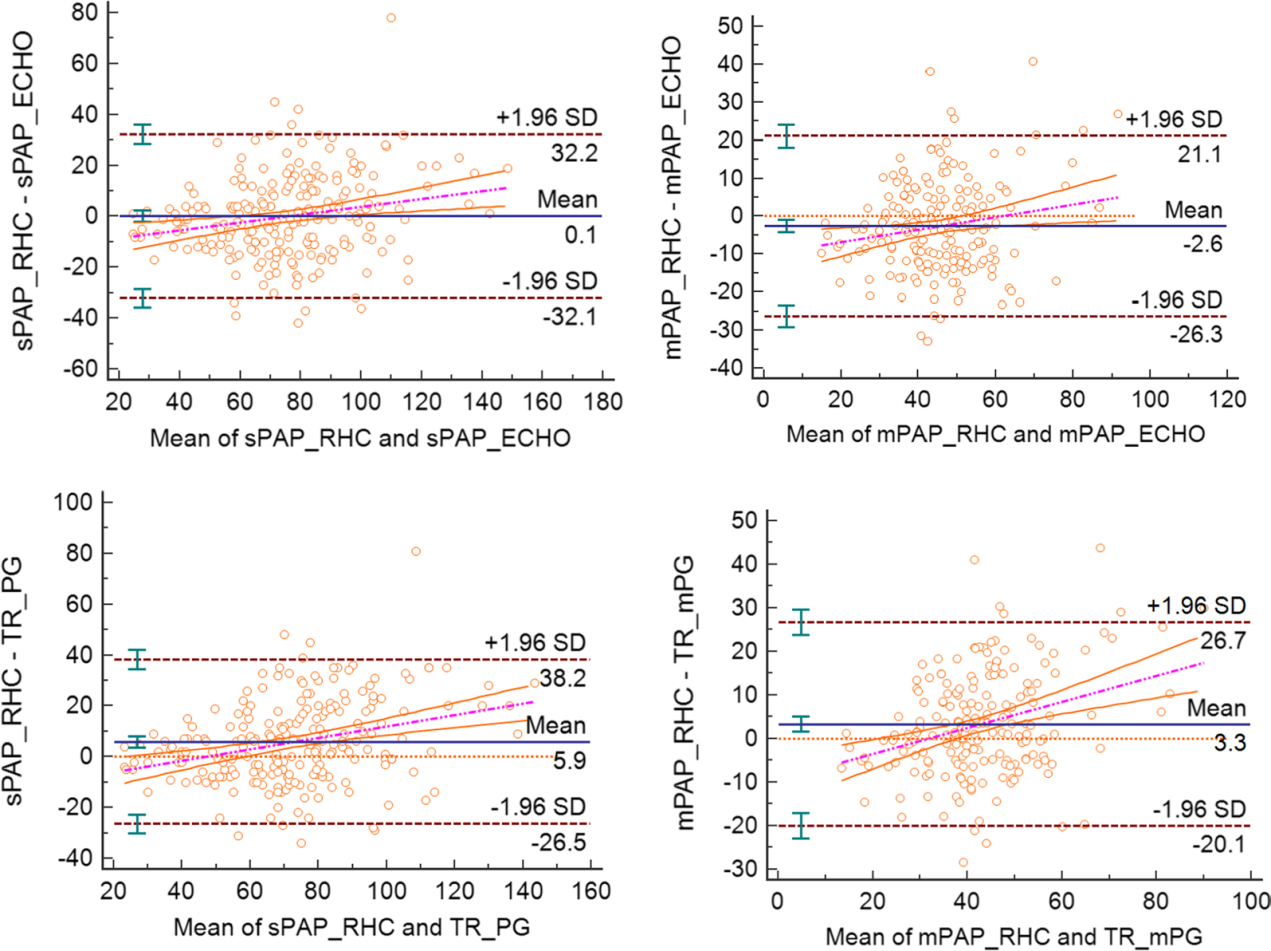
Bland–Altman plot showing the relationship between invasively determined parameters with TR derived parameters

All of the TR-derived parameters, including TR Vmax, TR-PG, TR-mPG, mPAP_ECHO_, and sPAP_ECHO_, showed a positive correlation with related RHC results (Fig. 3). sPAP_ECHO_ had the highest correlation coefficient (r = 0.782, *P* < 0.001). Bland–Altman analysis demonstrated low bias between RHC and echocardiographic results, with wide limits of agreements (Fig. 4). The bias of sPAP_ECHO_ (mean bias = 0.1 mm Hg; 95% limits of agreement: −32.1 to +32.2 mm Hg) was lower than that of TR-PG (mean bias = 5.9 mm Hg; 95% limits of agreement: −26.5 to +38.2 mm Hg). The mean deviations of mPAP_ECHO_ and TR-mPG from mPAP_RHC_ were −2.6 mm Hg (95% limits of agreement: −26.3 to +21.1 mm Hg) and 3.3 mm Hg (95% limits of agreement: −20.1 to +26.7 mm Hg), respectively.

### Performance of different TR methods for predicting PH

The ROC analysis showed that sPAP_ECHO_ had better predictive efficiency and sensitivity for determining the possibility of PH than other TR-related methods, including TR Vmax, TR-PG, TR-mPG, and mPAP_ECHO_ (Table 3), but their differences were not significant (*P* > 0.05). Using Youden index quantification, the optimal cutoff value for our cohort was 49.5 mm Hg for the sPAP_ECHO_ method with a sensitivity of 94.9% and a specificity of 85.7%.

**Table 3:** Receiver operating characteristic curve analysis of DE parameters for detecting PH (mPAP ≥ 25 mmHg)

	AUC	Cut-off value	Sensitivity (%)	Specificity (%)	Accuracy (%)	PPV (%)	NPV (%)
sPAP_ECHO_	0.981	49.5 mmHg	94.9	85.7	94.0	98.4	64.3
TR Vmax	0.977	350.0 cm/s	91.9	90.5	91.7	98.9	54.3
TR-PG	0.978	46.5 mmHg	94.4	85.7	93.6	98.4	62.1
mPAP_ECHO_	0.956	30.6 mmHg	94.4	84.6	93.7	98.8	52.4
TR-mPG	0.945	27.6 mmHg	92.7	84.6	92.2	98.8	45.8

*PPV* Positive predictive value; *NPV* Negative predictive value; *sPAP*_*ECHO*_: pulmonary systolic pressure estimated by echocardiography; *TR Vmax*: maximum velocity of tricuspid regurgitation; *TR-PG* tricuspid regurgitation pressure gradient; *mPAP*_*ECHO*_ mean pulmonary artery pressure estimated by echocardiography; *TR-mPG* tricuspid regurgitation mean pressure gradient

### Factors affecting the accuracy of sPAP_ECHO_ estimation

There were 79 patients (36.2%) in the overestimated group, 81 patients (37.2%) in the accurate group, and 58 patients (26.6%) in the underestimated group. sPAP_RHC_ was divided into three levels according to its tertiles (63 mm Hg, 85 mm Hg). The low-level group was defined sPAP_RHC_ less than 63 mm Hg. Patients with sPAP_RHC_ between 63 mm Hg and 85 mm Hg were considered the medium-level group, while patients with sPAP_RHC_ higher than 85 mmHg were classified as the high-level group. The univariate ordinal analysis demonstrated that RV WT, FAC, TR signal quality, sPAP_RHC_ level, RAP, PVR, PAWP, and mPAP were associated with the inaccuracy of sPAP_ECHO_ estimation (Table 2). After multivariate ordinal regression analysis, we found that TR signal quality, PAWP, and sPAP_RHC_ level significantly affected the accuracy of sPAP_ECHO_ (*P* < 0.05). Relative to good signal quality, the OR values of medium and poor signal quality were 0.26 (95% CI: 0.14, 0.48) and 0.23 (95% CI: 0.07, 0.73), respectively. Compared with high sPAP_RHC_ level, the OR values of low and medium sPAP_RHC_ levels were 21.56 (95% CI: 9.57, 48.55) and 5.13 (95% CI: 2.55, 10.32), respectively. The OR value of PAWP was 0.94 (95% CI: 0.89, 0.99). In contrast, TR severity and RV systolic function parameters (such as TAPSE, S’, and FAC) did not remain in the final equation.

## Discussion

Key findings of our study are as follows: (1) All TR-related methods, including sPAP_ECHO_, have comparable and good efficiency in PH screening. (2) The assessment of sPAP_ECHO_ would be more reliable after taking TR signal quality, sPAP_RHC_ levels, and PAWP into account.

### Performance of sPAP_ECHO_ in PH screening

In our study, sPAP_ECHO_ showed comparable efficiency to other TR-related methods in PH screening. Compared with mPAP_ECHO_, sPAP_ECHO_ is more convenient to measure. As a derived variable of TR Vmax, sPAP_ECHO_ did not amplify measurement errors in assessing pulmonary artery pressure as indicated by the current guidelines; on the contrary, it showed better sensitivity while maintaining similar specificity. Relative to TR Vmax, TR-PG, and TR-mPG, sPAP_ECHO_ contains more information from RAP, which may account for its better accuracy and lower bias. RAP elevates with the increase of RV overload [9], so it is an important measurement that provides heart failure and prognostic information [10]. Hellenkamp’s study [2] on mPAP_ECHO_ also supported that RAP is of additional diagnostic value in predicting PH. Compared with mPAP_ECHO_, sPAP_ECHO_ has the advantage of being simple and convenient. Taken together, sPAP_ECHO_ can be a convenient and effective measurement for clinical application in PH screening.

### Reasons for the inaccuracy in sPAP_ECHO_ estimation

First, our findings confirmed previous reports that the TR signal quality would affect the accuracy of sPAP_ECHO_ [11]. Poor signal quality leads to the underestimation of sPAP_ECHO_, because interpretation error of peak velocity is further amplified by the square of the Bernoulli equation. We also found that good signal quality could bring overestimation of sPAP_ECHO_ for some cases. In our cohort, sPAP_ECHO_ was still overestimated in 41% of the patients who obtained good signal quality of TR. After further analysis, we found that the lower sPAP_RHC_ level and PAWP were significantly associated with the overestimation of sPAP_ECHO_ in patients with good signal quality. This phenomenon suggests that we cannot simply rely on good signal quality, and attention should also be paid to sPAP_RHC_ level and PAWP because they both affect the accuracy of sPAP_ECHO_.

Second, as for the effect of sPAP_RHC_ level on the accuracy of sPAP_ECHO_, Groh et al. [12] found that echocardiography inaccurately estimated right ventricular pressure in children with elevated right heart pressure. Our results provided further evidence that sPAP_ECHO_ tends to be underestimated at a high sPAP_RHC_ level. We assumed that the coupling mechanism between RV contractility and its load may account for this phenomenon. When sPAP_RHC_ mildly elevates during the initial phase of PH, RV coupling could be maintained by enhanced RV contractility [13, 14], and the estimation of sPAP_RHC_ by DE is relatively reliable. However, as PH progresses and RV uncoupling occurs, CO would decrease and RV preload would increase, along with elevated RAP, so the right atrioventricular pressure gradient would decrease, and DE would underestimate sPAP_RHC_. sPAP_RHC_ level may affect the accuracy of sPAP_ECHO_ through the coupling mechanism between RV contractility and its load. This finding suggests that we should synthesize more echocardiographic signs when evaluating the efficacy of PH, because the decrease in sPAP_ECHO_ at this time is not necessarily a result of disease improvement, but may also be a sign of underestimation of sPAP_ECHO_ caused by RV decoupling.

Third, we found that echocardiography tended to underestimate pulmonary artery pressure when PAWP increased. We speculated that the underestimation of sPAP_ECHO_ due to the higher PAWP may be related to the lower threshold in post-capillary PH patients. Amsallem et al. [15] found that higher PAWP was associated with lower sPAP_ECHO_ threshold for PH diagnosis, which is consistent with our findings. The optimal cutoff value of our cohort was 49.5 mm Hg, which is higher than the cutoff values in previous studies that had focused on post-capillary PH patients with higher PAWP [16, 17]. Pre-capillary PH patients with lower PAWP accounted for 85.8% of the cohort, which may explain this phenomenon. Finkelhor et al. [18] also found that PAWP had a strong inverse correlation with the difference between sPAP_RHC_ and sPAP_ECHO_. They speculated that elevated left atrial pressure can be transmitted to the right atrium via the shared interatrial septum as well as through pericardial constraint and limit TR velocities, thereby also affecting the accuracy of sPAP_ECHO_. Until now, the mechanism by which PAWP affects the accuracy of sPAP_ECHO_ is still unclear, so more multicenter studies are needed to validate this deduction. Based on the above findings, we think that the accuracy of sPAP_ECHO_ would be improved if combined with the assessment of left ventricular filling pressure by echocardiography. Although RHC is the gold standard for PAWP or left ventricular filling pressure, whether PAWP is elevated can be assessed by indirect signs of echocardiography, such as the ratio of mitral E peak velocity and averaged e’velocity (E/e'm ratio), TR Vmax, and left atrium volume index. Echocardiologists can synthesize such information to determine whether patients have PAWP elevation, to assess the sPAP_ECHO_ and the possibility of PH more reasonably.

**Fig. 5:**
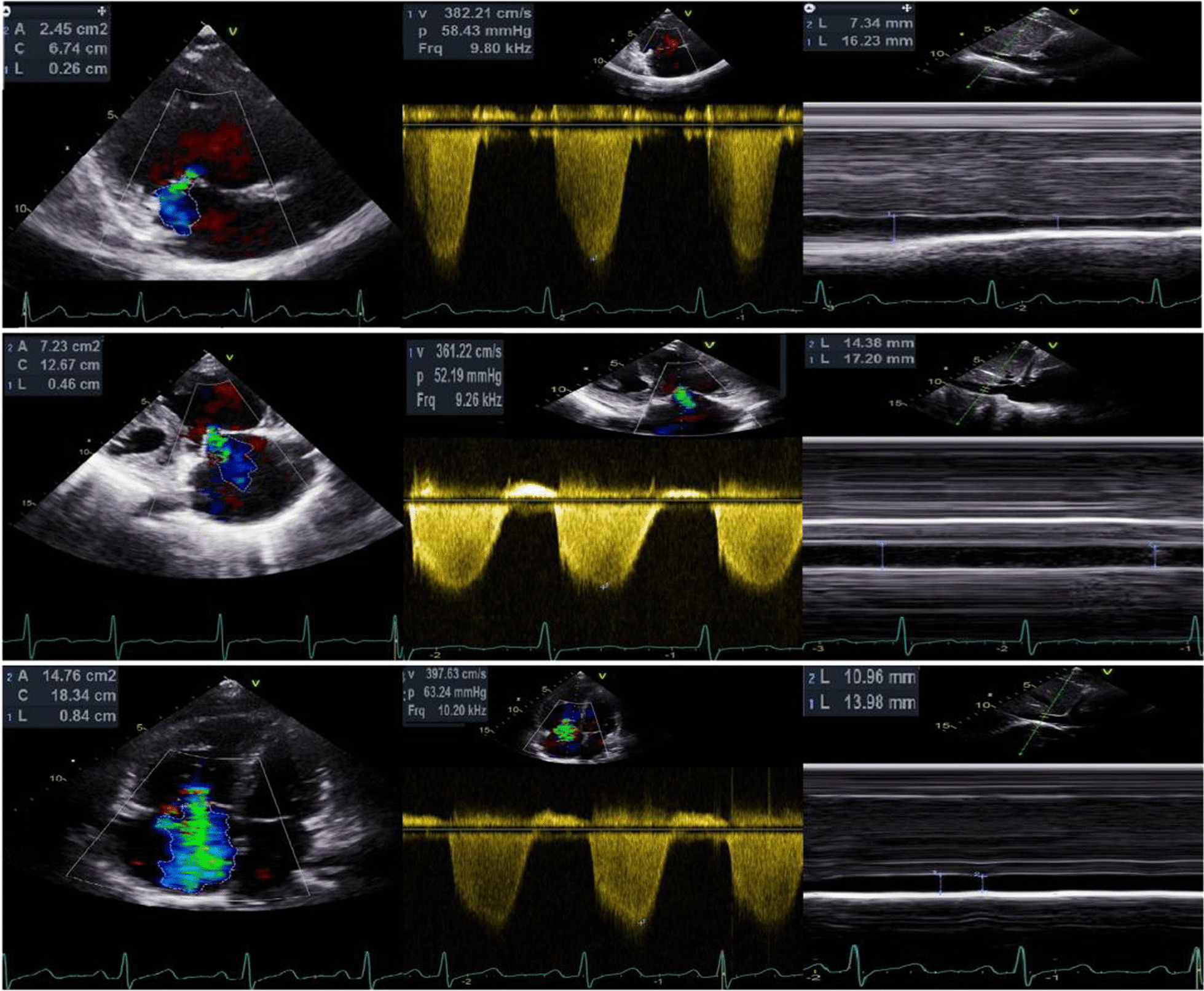
Examples of different severity of TR with good signal quality and accurate sPAP_ECHO_. The upper image presents a 40 years old female with mild TR whose sPAP_ECHO_ and sPAP_RHC_ were 59 and 61 mmHg, respectively. The medium image shows a 50 years old female with moderate TR whose sPAP_ECHO_ and sPAP_RHC_ were 60 and 60 mmHg, respectively. The lower image demonstrates a 34 years old female with severe TR whose sPAP_ECHO_ and sPAP_RHC_ were 71 and 73 mmHg, respectively

Furthermore, there is no consensus as to how TR severity would interfere with the accuracy of the sPAP_ECHO_. Hioka et al. [19] reported that echocardiography increasingly overestimated the TR PG with the advance of TR severity, as was theoretically predicted by the pressure recovery phenomenon associated with the laminar regurgitant flow. However, Parasuraman et al. [20] reported that severe TR could cause equalization of right atrial and ventricular pressures, which may cause the TR Doppler envelope to be cut short, thereby leading to underestimation of sPAP_ECHO_. Our study differed from other studies in that the TR severity did not significantly affect the accuracy of sPAP_ECHO_. It should be noted that only 9.6% of patients in our cohort had severe TR, which was in line with the actual clinical situation that severe TR only appears in the minority of patients. However, in patients with mild or moderate TR, we could also obtain good signal quality and estimate sPAP_ECHO_ appropriately (Fig. 5). TR severity was also affected by RV contractility and dimension. Thus, the overall impact of TR severity on the accuracy of sPAP_ECHO_ is not as significant as that of TR signal quality.

We found that none of the RV systolic parameters had a significant impact on the accuracy of sPAP_ECHO_. Theoretically, RV systolic function would gradually decrease [21], but RV can remain coupled for the large increase in load by increasing contractility until heart failure [13]. Therefore, RV systolic parameters are relatively stable before the end-stage of PH. In addition, the heart movement and measurement angle dependence also affect the accuracy of the relevant parameters. Although RV systolic parameters had clinical significance for the assessment of PH, they did not have a significant effect on the accuracy of sPAP_ECHO_.

Finally, incorporating TR signal quality, sPAP_RHC_ level, and PAWP into the assessment of sPAP_ECHO_ would improve its accuracy and avoid overly invasive examination.

### Limitations

This study has several limitations. First, this was a retrospective study with a small sample size. Although 90.5% of our patients had PH, and in 47.7% of them, PH was due to chronic pulmonary thromboembolism, the sample size of other types of PH was relatively small. Thus, we could not give specific suggestions for each type of PH. Second, we included patients who had undergone RHC and echocardiography within 7 days due to the restriction of clinical actual conditions. However, the average interval time was 3 days in this study, and the majority of patients had pre-capillary PH, which indicated that the patients’ hemodynamics was relatively stable and did not change dramatically during this short time. Furthermore, contrast microbubbles were not adopted to enhance the tricuspid regurgitation jet for patients with mild regurgitation or poor signal quality. Finally, the single-center nature of the present study limited generalization.

## Conclusions

In this study, we found that all TR-related methods, including sPAP_ECHO_, had comparable and good efficiency in PH screening. To make the assessment of sPAP_ECHO_ more accurate, attention should be paid to TR signal quality, sPAP_RHC_ level, and PAWP.

## Data Availability

The datasets used and analyzed during the current study are available from the corresponding author on reasonable request.

## Abbreviations

PH: Pulmonary hypertension
RHC: Right heart catheterization
DE: Doppler echocardiography
TR: Tricuspid regurgitation
sPAP_RHC_: Pulmonary artery systolic pressure measured by RHC
sPAP_ECHO_: Pulmonary artery systolic pressure estimated by DE
mPAP: Mean pulmonary artery pressure measured by RHC
mPAP_ECHO_: Mean pulmonary artery pressure estimated by DE
PAWP: Pulmonary artery wedge pressure
RAP: Right atrial pressure
CO: Cardiac output
TR PG: TR pressure gradient
TR-mPG: TR mean pressure gradient
PVR: Pulmonary vascular resistance
RV: Right ventricle
RV WT: RV wall thickness
TAPSE: Tricuspid annular plane systolic excursion
S’: Systolic annular tissue velocity of the lateral tricuspid annulus
FAC: RV Fractional area change
IVC: Inferior vena cava
